# Global research status and hot trends in stem cells therapy for Intervertebral disc degeneration: A bibliometric and clinical study analysis

**DOI:** 10.3389/fphar.2022.873177

**Published:** 2022-08-08

**Authors:** Nan Wang, Shuang Chen, Xiaoyu Zhang, Zhipeng Xi, Xiaoyang Fang, Congyang Xue, Jingchi Li, Lin Xie

**Affiliations:** Department of Spine Surgery, Affiliated Hospital of Integrated Traditional Chinese and Western Medicine, Nanjing University of Chinese Medicine, Nanjing, China

**Keywords:** bibliometrics, intervertebral disc degeneration, stem cells, visualization research, treatment

## Abstract

**Background:** Stem cells (SCs) therapy for intervertebral disc degeneration (IDD) has been studied for nearly 20 years and it is an important part of regenerative medicine and tissue engineering research, as well as a current research hotspot and challenge. Although the volume of literature has shown an annual growth trend, there is no literature available for bibliometric and clinical analysis of the content of multiple databases in this field.

**Methods:** The articles were obtained from the WOSCC, Scopus, Pubmed, and ClinicalTrials on 27 December 2021. Three scientometric software (VOSviewer 1.6.17, CiteSpace 5.8.R.1 and Scimago Graphica) were used to perform bibliometric and knowledge-map analysis.

**Results:** We included 867 articles from WOSCC, 716 articles from Scopus and 6 clinical studies from ClinicalTrials for literature analysis. Our results showed that China was the country with the highest number of publications, with the United States (US) being the leader in terms of international collaborations and the number of citations. Sakai D, Grad S and Hoyland JA had made outstanding contributions for their high productivity and the quality articles. *Spine* was the most published and most cited journal, in addition to *Spine Journal* and *Biomaterials*, which were also more authoritative journals and had received high citations. All of them had received high citations. Keyword co-occurrence studies suggested that the current hotspots were in mechanistic studies, including inflammation, apoptosis, exosome, autophagy, and others. Some studies had also investigated tissue-engineered scaffolds of SCs to better repair degenerated discs. Clinical studies were relatively scarce. Direct injection of Mesenchymal Stem Cells (MSCs) into degenerated discs for the treatment of Degenerative disc disease (DDD) was the current direction of research.

**Conclusion:** This study demonstrates the global research hotspots, trends and clinical use of SCs in the treatment of IDD. It can help scholars to quickly understand the current status and hotspots of research in this field, and also provide some guidance and reference for those who are currently researching in this area.

## Introduction

Intervertebral disc degeneration (IDD) is a chronic degenerative disease that causes an imbalance in the internal environment of the disc and affects the overall mobility of the spine (Risbud and Shapiro, 2014). It is widely recognised that IDD is a primary source of low back pain (LBP), and in severe cases causes disability (Cheung et al., 2009; de Schepper et al., 2010). It imposes a great financial load on individuals, households and even upon society (Luoma et al., 2000). The treatment of LBP is currently divided into conservative and surgical treatments (Chen et al., 2016). Unfortunately, these treatments provide partial relief but not address the underlying pathology of IDD. However, the rise of cellular therapy in recent years has made disc repair and regeneration possible (Harfe, 2022). Various studies have shown that Stem Cells (SCs) can target degenerated discs in a variety of ways, making them a particularly attractive treatment option, but the exact mechanisms are still being investigated (Hohaus et al., 2008; Krut et al., 2021).

Bibliometrics is a tool that statistical and mathematical methods are used to evaluate trends and hotspots in the field of published literature and studies (Ozsoy and Demir, 2018). Different from traditional citation statistics, it can account for links between documents and recognize knowledge structures and rising trends (Wang et al., 2021). Because of its distinctive advantages, it has become especially important in evaluating hotspots in literature and developing further projects (Chen et al., 2020). This study provides a systematic summary of the research on SCs in IDD, looking for its research frontiers, hotspots and trends, and reviewing the characteristics of clinical trials, summarising their development process and visualising them, which can provide a reference for further research in the future.

## Materials and methods

### Data sources and search strategies

**FIGURE 1:**
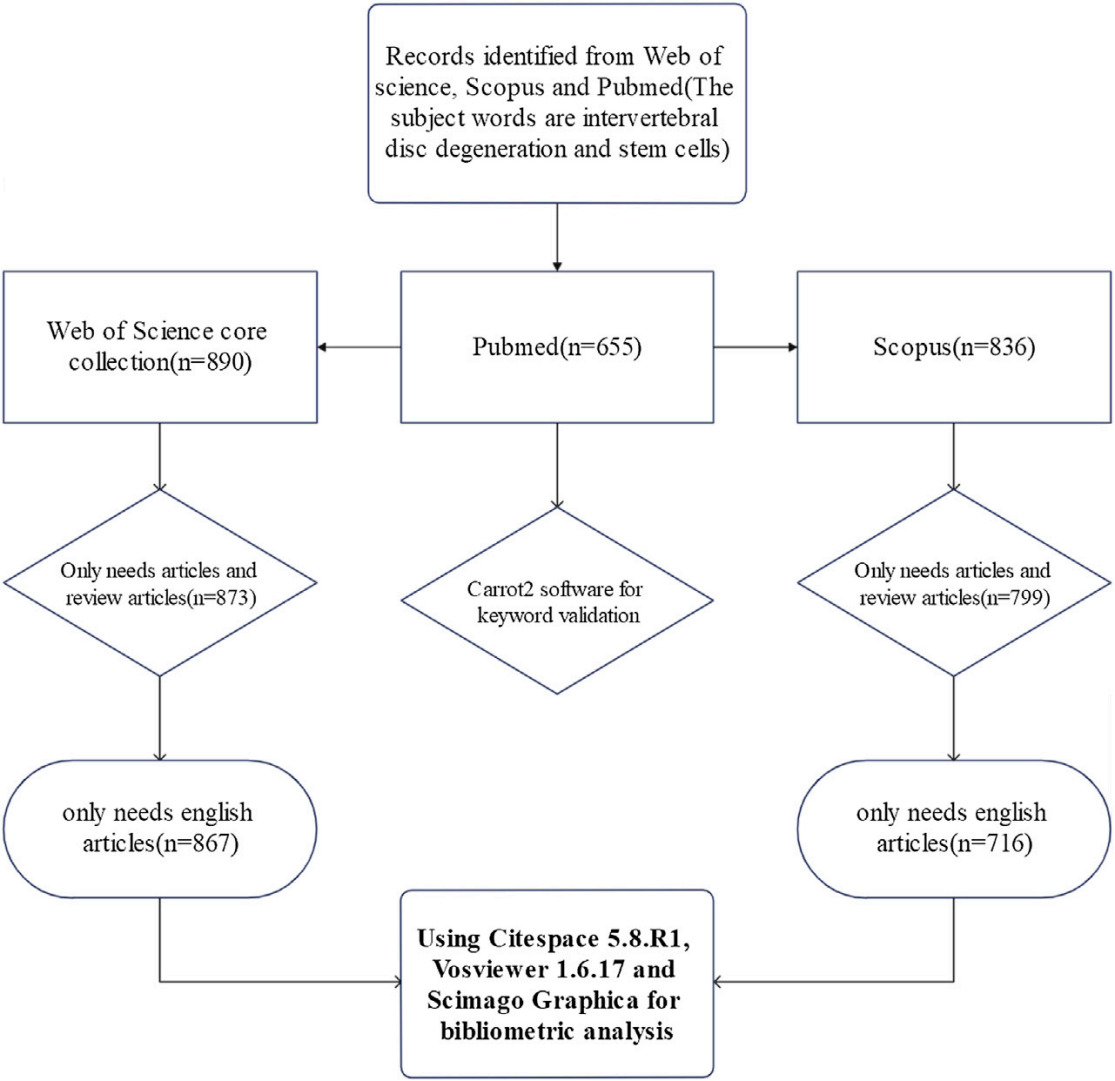
The flow chart of search strategy about SCs therapy for IDD. Full search completed on 27 December 2021. 890 articles were searched from WOSCC and 836 studies were identified from Scopus. We only needed reviews and original articles and chose English as the language type. Eventually, 867 articles in WOSCC and 716 articles in Scopus were included in the analysis.

The bibliometric search was conducted in the Web of science, Scopus and Pubmed database on 27 December 2021. The search strategy can be seen in Figure 1. In the Web of Science core collection (WOSCC) and Scopus databases, the publication type was limited to “article” and “review” and the language was restricted to English. In the Pubmed database, there was no restriction on the search results, since it is mainly used to verify keywords. Topic words were obtained from PubMed’s MESH. Using the Scopus database as an example, the search strategy was shown below: (TITLE-ABS-KEY (stem AND cells) AND TITLE-ABS-KEY (intervertebral AND disc AND degeneration)) AND (LIMIT-TO (DOCTYPE, “ar”) OR LIMIT-TO (DOCTYPE, “re”)) AND (LIMIT-TO ( LANGUAGE, “English”)).

### Data collection

In the WOSCC, the results of the search were downloaded as “Full Record and Cited References” and the file format was “Plain Text” and “Windows UTF-8.” The former was processed and imported into bibliometric software for further analysis, while the latter was uploaded directly to the network for online analysis. In the Scopus, the files in “CSV” format were downloaded for subsequent analysis. The Carrot2 provided direct access to the clustering results for keywords in Pubmed (Fang et al., 2020), which allowed for some validation of the results from the previous 2 databases.

### Statistical analysis and visualization software

The WOSCC and Scopus databases included its own analysis function for authors, affiliations, countries, publication titles and year of publication. The downloaded data was imported into Microsoft Excel 2016 for statistical analysis. We adopted VOSviewer 1.6.17, CiteSpace 5.8.R.1 and Scimago Graphica to recognize the network maps of journals, authors, countries/regions, institutions, cited references, co-cited references and keywords. In addition, Citespace was used to depict dual-map overlays of journals and identify keywords with a strong burst strength for exploring emerging topics. The mapping of the world was done through an online website (http://lert.co.nz/map/).

### Clinical study analysis

The ClinicalTrials.gov website (https://www.clinicaltrials.gov/) was used to search for current clinical studies of SCs therapy for IDD. The disease was “Degenerative Disc Disease,” the intervention was “Stem Cells” and the status was all studies.

## Results

### Annual publications

**FIGURE 2:**
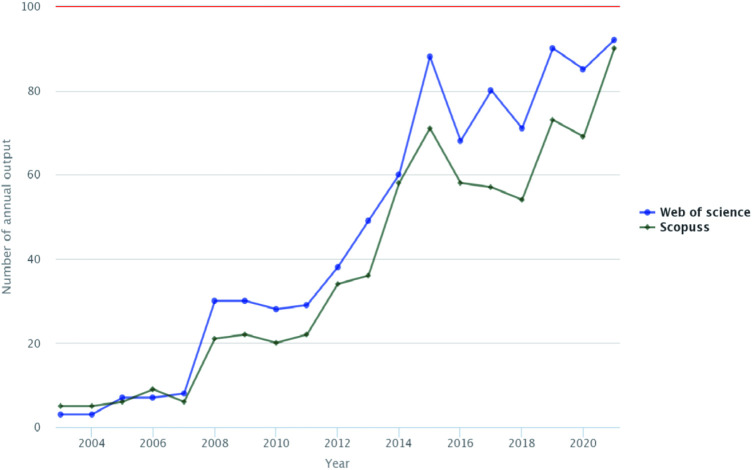
Annual publications of SCs therapy for IDD from 2003 to 2021. The trends can be split into three phases. First, 2003–2007, the initiation phase, is characterised by a slow rise; second, the rapid development phase, 2007–2015, is characterised by a continuous rise; third, the stable development phase, 2015–2021, is characterised by a circular rise. 2021 is also by far the most published year.

The included literature was published from 2003 to 2021, of which the most was this year. As can be seen from Figure 2, there was a significant trend in global literature over time. After the first publication in 2003, this field of study gained a lot of attention from researchers. 2010–2015 saw a linear increase in the number of articles in this field. 2016–2021 saw an upward spiral, and even though 2021 is not quite over yet, it has already become the highest ever in terms of the number of articles published.

### Country/region analysis and international cooperation

**FIGURE 3:**
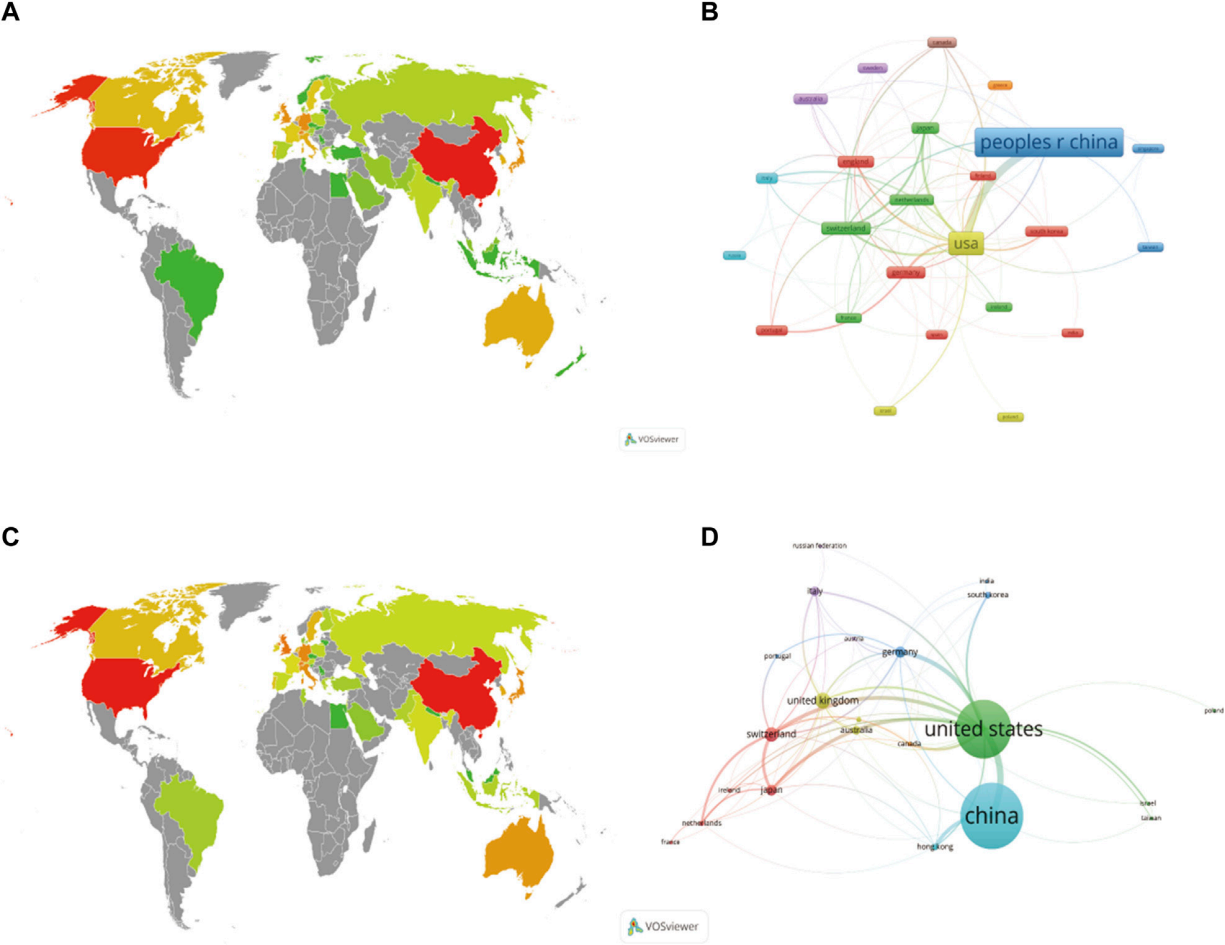
Global distribution of national publications in WOSCC **(A)** and Scopus **(C)**. The darker the colour, the greater the volume of literature, with both the China and US panels in red on the world map, indicating the highest volume of publications. Cooperation networks between countries/region in WOSCC **(B)** and Scopus **(D)**. Different colours represented different categories, while countries cooperated with each other by linking lines, with thicker connections implying closer cooperation. A node represented a region, with larger segments indicating more national issuance. The graph illustrated that although China has a higher volume of publications, the US cooperated more closely with other countries in international cooperation.

When the country/region contributions to the field were counted (Table 1), it was found that China was the most published country in both WOSCC (340) and Scopus (238), followed by the United States (239 and 212). However, in terms of citations, the US (9512 and 7727) outnumbered China (7132 and 4349), with a considerably higher average citation rate than any other country, and took first place. The top five countries in terms of publication volume were China, US, United Kingdom, Switzerland and Germany, although the order changed between the two databases (Figures 3A,C).

**TABLE 1:** Top 10 Countrys contributing to Publications in the WOSCC and Scoups database.

Rank	WOSCC			Scopus		
	Country (*n* = 46)	Documents (%)	Citations	Country (*n* = 42)	Documents (%)	Citations
1	China	340 (39.22)	7132	China	238 (33.24)	4349
2	United States	239 (27.57)	9512	United States	212 (29.61)	7727
3	Switzerland	66 (7.61)	3143	United Kingdom	57 (7.96)	2832
4	Germany	55 (6.34)	1660	Switzerland	52 (7.26)	2401
5	United Kingdom	54 (6.23)	2958	Germany	41 (5.73)	1240
6	Japan	50 (5.77)	3411	Japan	39 (5.45)	3309
7	Netherlands	37 (4.27)	1098	Italy	36 (5.03)	1319
8	Australia	31 (3.58)	921	Australia	32 (4.47)	905
9	Italy	31 (3.58)	1090	Hong Kong	25 (3.49)	1303
10	South Korea	30 (3.46)	705	South Korea	24 (3.35)	767

**FIGURE 4:**
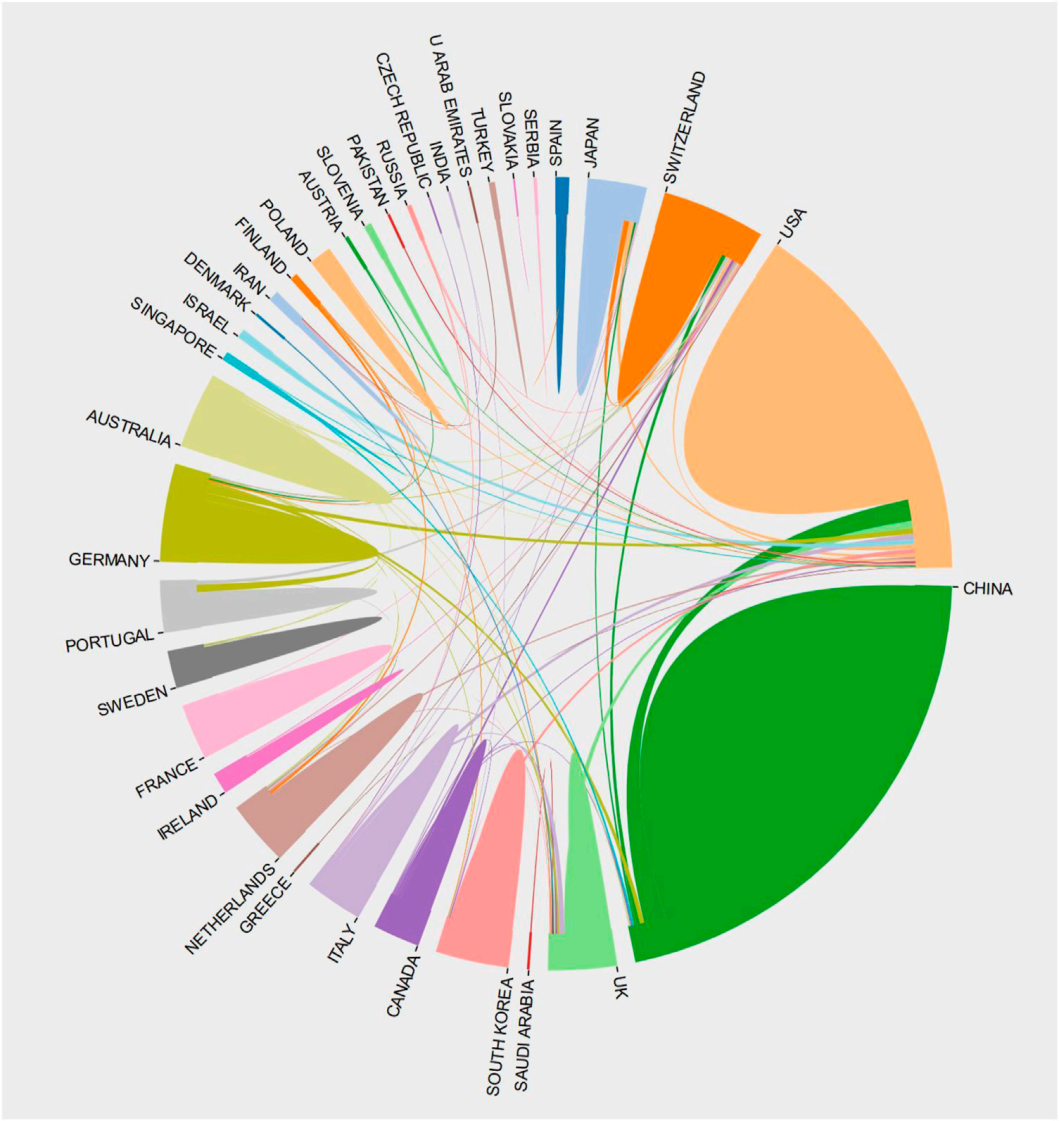
Inter-country cooperation chord chart display (take WOSCC as an example). The larger the area the greater the number of national publications, and the thicker the line the stronger the links. It can be seen that the Western countries, led by the US, were more closely linked to each other, and although China and the United States were closely linked, there was still a lack of multi-national cooperation with others.

By using the VOSviewer to visualise cooperation between countries, different colours represent different clusters and there is close cooperation between countries in each cluster, with the active countries in the WOSCC data clustered into 8 categories and the Scopus data clustered into 7 categories. The WOSCC results showed that England, Finland, Germany, India, Portugal, South Korea and Spain were grouped in cluster 1 and there was a strong link between them. China was placed in the third cluster and the United States in the fourth (Figure 3B). However, the Scopus data showed that Switzerland, Japan, the Netherlands, Ireland and France were in cluster 1, which indicated in red, and that there were strong links between them. The United States was in cluster 2, while China was in cluster 6 (Figure 3D). The results of the country collaboration analysis using the bibliometric online analysis platform indicated that the United States played an important role in the collaboration network, followed by Switzerland, and that although China had the highest number of publications, it still lacks collaboration with other countries and needs to strengthen international exchange in the future (Figure 4).

### Contributions of authors

A total of 3300 authors were found in WOSCC and 2607 in Scopus for this research area (Table 2). The three most published authors were Sakai D, Grad S and Hoyland JA, from Japan, Switzerland and the United Kingdom, respectively. In WOSCC, the top 10 authors published 27.68% of the total number, compared to 28.21% in Scopus. The most cited author was Sakai D, followed by Hoyland JA and Grad S. It was evident that these three authors had a high volume of publications with high citation rates and had a significant influential status in this field.

**TABLE 2:** Top 10 authors contributing to Publications in the WOSCC and Scoups database.

Rank	WOSCC			Scopus		
	Author (*n* = 3300)	Documents (%)	Citations	Author (*n* = 2607)	Documents (%)	Citations
1	Sakai D	35 (4.04)	1950	Grad S	27 (3.77)	1493
2	Grad S	30 (3.46)	1665	Sakai D	27 (3.77)	2723
3	Hoyland JA	28 (3.23)	1827	Hoyland JA	24 (3.35)	1817
4	Alini M	24 (2.77)	1258	Zhou Y	21 (2.93)	383
5	Richardson SM	22 (2.54)	1446	Richardson SM	20 (2.79)	1441
6	Zhou Y	22 (2.54)	457	Alini M	18 (2.51)	1372
7	Cheung KMC	20 (2.31)	669	Brisby H	18 (2.51)	681
8	Li FC	20 (2.31)	350	Benneker LM	16 (2.23)	713
9	Zhang L	20 (2.31)	175	Cheung KMC	16 (2.23)	593
10	Chen QX	19 (2.19)	308	Li FC	15 (2.09)	459

**FIGURE 5:**
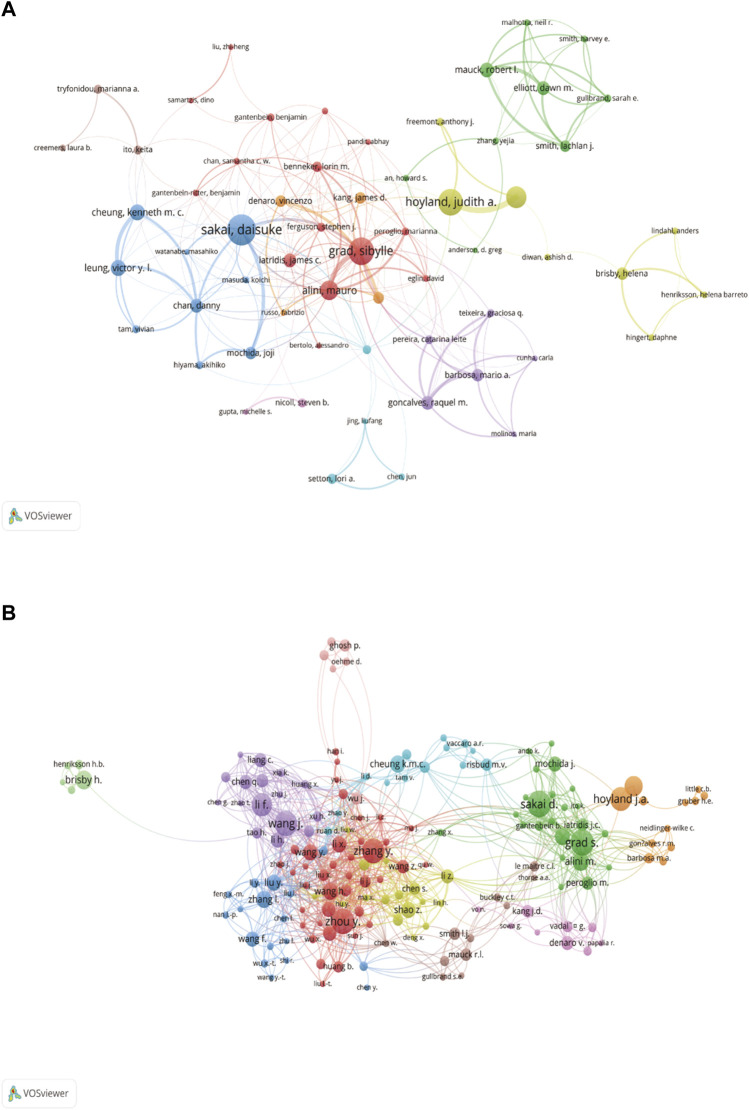
Cooperation networks between Authors in WOSCC **(A)** and Scopus **(B)**. Different colours represented different clusters, while authors cooperated with each other by linking lines, with thicker connections implying closer cooperation. A node represented an author, with larger segments indicating more publications. In WOSCC, key authors formed 9 clusters and 11 clusters were formed from Scopus.

A visual analysis of the authors’ collaborations was performed using the VOSviewer software. In WOSCC, the important authors were divided into 9 clusters (Figure 5A). The first cluster was represented by Grad S, Alini M, Benneker lM, among others, who formed a close collaborative network with each other. And Grad S had the highest total link strength of 84, which highlighted his importance in cluster 1. Cluster 2 was represented by Mauck, Rl and others, and cluster 3 was Sakai D with others.

However, in Scopus, important authors were divided into 11 clusters (Figure 5B), each indicated by a different color. In cluster 1, Chinese scholars represented by Zhou Y and Zhang Y were interconnected and formed a complex network, mostly from China, but lacking collaboration with internationally recognized experts. Grad S had the highest total link strength with a value of 127, and he was in cluster 2. The second and third ranked authors in terms of total link strength were Wang J and Li F respectively, both of whom were in cluster 5.

### Journal analysis

The results of both databases showed that *Spine* (IF = 3.47, Q1) and *Spine journal* (IF = 4.17, Q1) were the two most widely published journals in the field (Table 3). Although S*pine* was recognised as the most widely published journal in this research area, the most highly cited journal was *Biomaterials* (IF = 12.48, Q1). It was clear that *Biomaterials* was the more authoritative journal in the stem cell field.

**TABLE 3:** Top 10 Journals contributing to Publications in the WOSCC and Scoups database.

Rank	WOSCC			Scopus		
	Journals	Documents (%)	Citations	Journals	Documents (%)	Citations
1	Spine	41 (4.73)	2062	Spine	32 (4.47)	2385
2	Spine Journal	36 (4.15)	1223	Spine Journal	27 (3.77)	1143
3	European Cells And Materials	32 (3.69)	918	Journal Of Orthopaedic Research	25 (3.49)	1106
4	European Spine Journal	30 (3.46)	2161	Stem Cell Research And Therapy	21 (2.93)	469
5	Journal Of Orthopaedic Research	30 (3.46)	1253	Stem Cells International	21 (2.93)	381
6	Acta Biomaterialia	29 (3.35)	780	European Spine Journal	18 (2.51)	768
7	Biomaterials	29 (3.35)	2129	Biomaterials	17 (2.37)	1598
8	Tissue Engineering Part A	29 (3.35)	814	Acta Biomaterialia	16 (2.23)	565
9	Stem Cells International	22 (2.54)	346	Current Stem Cell Research And Therapy	16 (2.23)	294
10	Journal Of Tissue Engineering And Regenerative Medicine	19 (2.19)	538	European Cells And Materials	16 (2.23)	629

**FIGURE 6:**
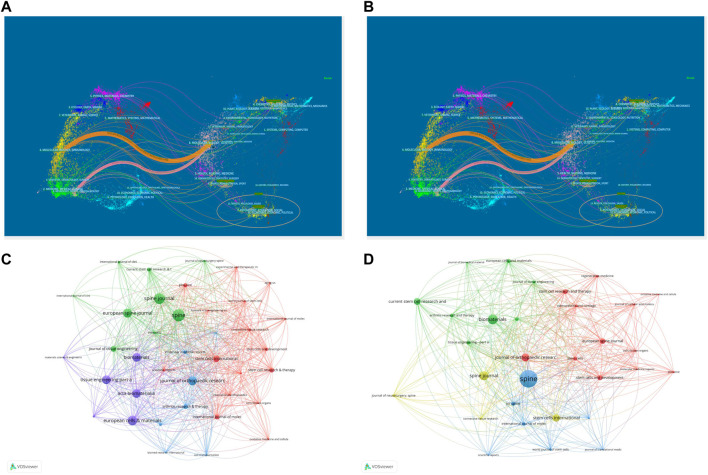
The dual-map overlay of journals in WOSCC **(A)** and Scopus **(B)**. The dual-map overlay of the journal showed a total of one pink primary citation path and one orange primary citation path. The journal network graph in WOSCC **(C)** and Scopus **(D)**. In WOSCC, key journals formed 4 clusters and 4 clusters were formed from Scopus.

The dual-map overlay of the journal showed a total of one pink primary citation path and one orange primary citation path (Figures 6A,B). The pink path represents that publications in Neurology/Sports/Ophthalmology journals cite publications in Molecular/Biology/Genetics journals. The orange path suggests that publications in Molecular/Biology/Immunology journals typically usually cite publications in Molecular/Biology/Genetics journals. The journal network graph showed that *Spine* had the highest total link strength in the network (WOSCC = 704, Scopus = 596), followed by *Biomaterials* (WOSCC = 660, Scopus = 331), indicating their important role in the field of journals (Figures 6C,D).

## Highly cited articles

Chan, B. P’s 2008 article in the *European Spine Journal* entitled “Scaffolding in tissue engineering: general approaches and tissue-specific considerations” was the most cited paper. It was a review and had a Norm. citations of 7.87. Apart from the first article in WOSCC, the top 5 highly cited articles were the same in both databases. This reinforced the importance of these articles in the field, and Sakai, D had made a significant contribution to this research area, with four of the top 6 highly cited publications in WOSCC and four of the top 5 in Scopus coming from him (Table 4). Citations are not a fully objective representation of the importance of literature, as it is influenced by duration. Norm. citations, on the other hand, exclude this interference. The high Norm. citations of an article, even if it does not have a high total number of citations, is proof of its importance. Of the two databases, a 2016 article by Richardson, SM entitled “Mesenchymal stem cells in regenerative medicine: Focus on articular cartilage and intervertebral disc regeneration” published in *Methods* received the highest Norm. citations. Although it was published late, it still received a lot of attention and obtained a high number of citations right after publication.

**TABLE 4:** Top 10 highly cited articles in the WOSCC and Scoups database.

Rank	Author	Title (WOSCC)	Journal	Year	Citations	Norm. citations
1	Chan, B. P	Scaffolding in tissue engineering: general approaches and tissue-specific considerations	European Spine Journal	2008	748	7.8682
2	Sakai, D	Transplantation of mesenchymal stem cells embedded in Atelocollagen [(R)] gel to the intervertebral disc: a potential therapeutic model for disc degeneration	Biomaterials	2003	304	2.2353
3	Orozco, L	Intervertebral Disc Repair by Autologous Mesenchymal Bone Marrow Cells: A Pilot Study	Transplantation	2011	279	4.7041
4	Sakai, D	Regenerative effects of transplanting mesenchymal stem cells embedded in atelocollagen to the degenerated intervertebral disc	Biomaterials	2006	274	1.7233
5	Sakai, D	Differentiation of mesenchymal stem cells transplanted to a rabbit degenerative disc model - Potential and limitations for stem cell therapy in disc regeneration	Spine	2005	256	2.5747
6	Sakai, D	Exhaustion of nucleus pulposus progenitor cells with ageing and degeneration of the intervertebral disc	Nature Communications	2012	248	4.9995
7	Zhao, CQ	The cell biology of intervertebral disc aging and degeneration	Ageing Research Reviews	2007	246	2.5997
8	Crevensten, G	Intervertebral disc cell therapy for regeneration: Mesenchymal stem cell implantation in rat intervertebral discs	Annals of Biomedical Engineering	2004	238	2.0938
9	Richardson, SM	Mesenchymal stem cells in regenerative medicine: Focus on articular cartilage and intervertebral disc regeneration	Methods	2016	225	8.3062
10	Richardson, SM	Intervertebral disc cell-mediated mesenchymal stem cell differentiation	Stem Cells	2006	224	1.4088
11	Sakai D	Transplantation of mesenchymal stem cells embedded in Atelocollagen^®^ gel to the intervertebral disc: A potential therapeutic model for disc degeneration	Biomaterials	2003	352	3.5341
12	Orozco L	Intervertebral disc repair by autologous mesenchymal bone marrow cells: A pilot study	Transplantation	2011	318	5.9949
13	Sakai D	Regenerative effects of transplanting mesenchymal stem cells embedded in atelocollagen to the degenerated intervertebral disc	Biomaterials	2006	310	3.0829
14	Sakai D	Differentiation of mesenchymal stem cells transplanted to a rabbit degenerative disc model: Potential and limitations for stem cell therapy in disc regeneration	Spine	2005	297	3.0884
15	Sakai D	Exhaustion of nucleus pulposus progenitor cells with ageing and degeneration of the intervertebral disc	Nature Communications	2012	261	4.5884
16	Richardson S.M	Intervertebral disc cell-mediated mesenchymal stem cell differentiation	Stem Cells	2006	259	2.5757
17	Richardson S.M	Mesenchymal stem cells in regenerative medicine: Focus on articular cartilage and intervertebral disc regeneration	Methods	2016	237	7.9228
18	Sakai D	Stem cell therapy for intervertebral disc regeneration: Obstacles and solutions	Nature Reviews Rheumatology	2015	227	6.1119
19	Mwale F	Distinction between the extracellular matrix of the nucleus pulposus and hyaline cartilage: A requisite for tissue engineering of intervertebral disc	European Cells and Materials	2004	227	2.4047
20	Risbud M.V	Evidence for skeletal progenitor cells in the degenerate human intervertebral disc	Spine	2007	221	2.5402

**FIGURE 7:**
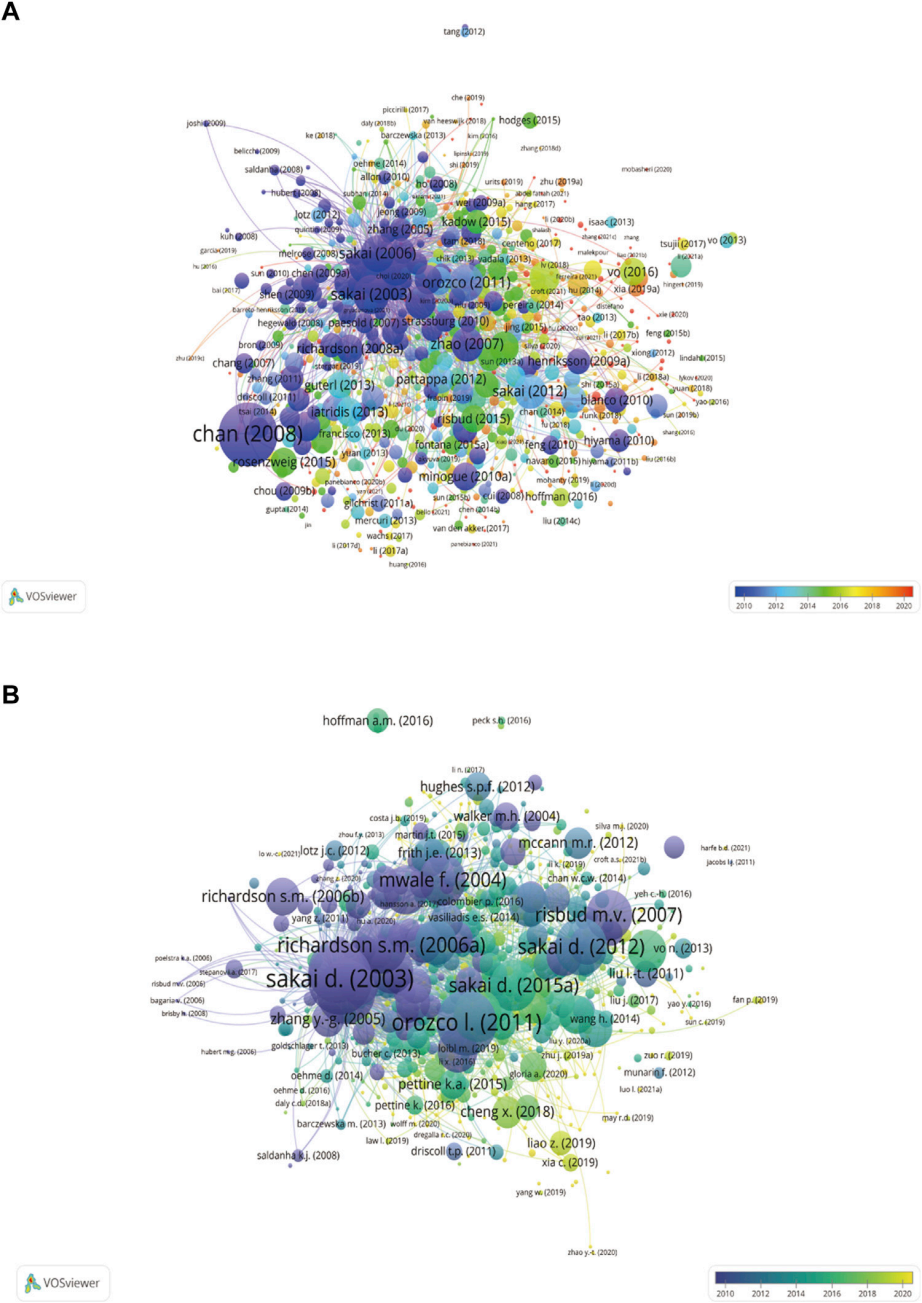
Overlay visualization plots of cited literature in WOSCC **(A)** and Scopus **(B)**. The different points represented different documents, which formed a complex network between them. The more frequently cited documents were represented as larger points in the graph. The different colours of the dots represented different publication periods. For example, in WOSCC, chan (2008) was the largest point, indicating that it was the most cited document, while its blue colour indicated that it was published earlier in the whole network. What’s more, in Scopus, cheng x (2018) was the brighter point, indicating that it was published later.

Highly cited articles were presented visually *via* VOSviewer software. The different points represented different documents, which formed a complex network between them. The more frequently cited documents were represented as larger points in the graph. The different colours of the dots represented different publication periods. For example, in WOSCC (Figure 7A), chan (2008) was the largest point, indicating that it was the most cited document, while its blue colour indicated that it was published earlier in the whole network. What’s more, in Scopus (Figure 7B), cheng x (2018) was the brighter point, indicating that it was published later.

### Co-citation articles

The top 10 co-cited references in the WOSCC and Scopus database were shown in Table 5. A total of five of the top 10 co-citations in both databases were identical, indicating the stronger linkage of these five to research in the field. Sakai D’s 2006 article in *Biomaterials*, entitled “Regenerative effects of transplanting mesenchymal stem cells embedded in atelocollagen to the degenerated intervertebral disc,” had the highest total link strength in the co-citation analysis. This article deserves to be read and studied carefully.

**TABLE 5:** Top 10 Co-Citation articles in the WOSCC and Scoups database.

Rank	Author	Title (WOSCC)	Journal	Year	Citations	Total link strength
1	Sakai D	Transplantation of mesenchymal stem cells embedded in Atelocollagen^®^ gel to the intervertebral disc: A potential therapeutic model for disc degeneration	Biomaterials	2003	174	4109
2	Sakai D	Regenerative effects of transplanting mesenchymal stem cells embedded in atelocollagen to the degenerated intervertebral disc	Biomaterials	2006	166	4344
3	Risbud M.V	Differentiation of mesenchymal stem cells towards a nucleus pulposus-like phenotype *in vitro*: implications for cell-based transplantation therapy	Spine	2004	159	3660
4	Sakai D	Differentiation of mesenchymal stem cells transplanted to a rabbit degenerative disc model: Potential and limitations for stem cell therapy in disc regeneration	Spine	2005	150	4015
5	Orozco L	Intervertebral disc repair by autologous mesenchymal bone marrow cells: A pilot study	Transplantation	2011	130	3512
6	Sakai, D	Exhaustion of nucleus pulposus progenitor cells with ageing and degeneration of the intervertebral disc	Nature Communications	2012	124	3234
7	Crevensten, G	Intervertebral disc cell therapy for regeneration: Mesenchymal stem cell implantation in rat intervertebral discs	Annals of Biomedical Engineering	2004	123	3353
8	Richardson, SM	Intervertebral disc cell-mediated mesenchymal stem cell differentiation	Stem Cells	2006	122	3042
9	Hiyama, A	Transplantation of mesenchymal stem cells in a canine disc degeneration model	Journal Of Orthopaedic Research	2003	119	2758
10	Hiyama, A	Transplantation of mesenchymal stem cells in a canine disc degeneration model	Journal Of Orthopaedic Research	2008	118	3174
11	Sakai D	Regenerative effects of transplanting mesenchymal stem cells embedded in atelocollagen to the degenerated intervertebral disc	Biomaterials	2006	451	36
12	Sakai D	Transplantation of mesenchymal stem cells embedded in Atelocollagen^®^ gel to the intervertebral disc: A potential therapeutic model for disc degeneration	Biomaterials	2003	367	31
13	Buckwalter, JA	aging and degeneration of the human intervertebral disc	Spine	1995	323	27
14	Risbud M.V	Differentiation of mesenchymal stem cells towards a nucleus pulposus-like phenotype *in vitro*: implications for cell-based transplantation therapy	Spine	2004	327	26
15	Andersso GB	Epidemiological features of chronic low-back pain	Lancet	1999	189	26
16	Aguiar, DJ	Notochordal cells interact with nucleus pulposus cells: regulation of proteoglycan synthesis	Experimental cell research	1999	343	25
17	Richardson, SM	Intervertebral disc cell-mediated mesenchymal stem cell differentiation	Stem Cells	2006	311	25
18	Le Maitre, CL	The role of interleukin-1 in the pathogenesis of human intervertebral disc degeneration	Arthritis Research andTherapy	2005	299	24
19	Orozco, L	Intervertebral Disc Repair by Autologous Mesenchymal Bone Marrow Cells: A Pilot Study	Transplantation	2011	261	24
20	Sakai D	Differentiation of mesenchymal stem cells transplanted to a rabbit degenerative disc model: Potential and limitations for stem cell therapy in disc regeneration	Spine	2005	306	23

**FIGURE 8:**
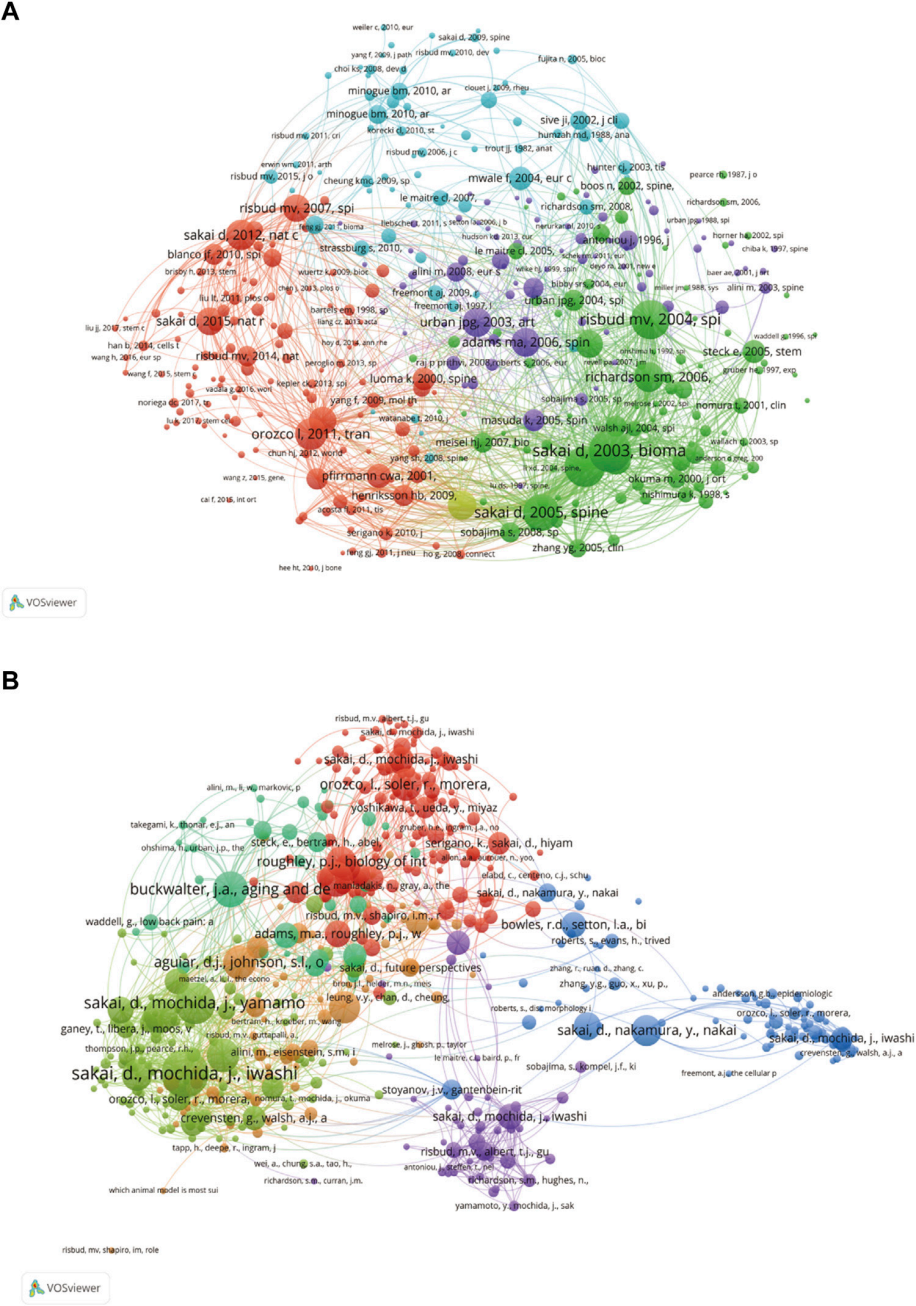
The co-citations network graph in WOSCC **(A)** and Scopus **(B)**. In WOSCC, co-citations formed 5 clusters and 6 clusters were formed from Scopus. Different colours represented different clusters. The size of a node represented how many citations the document has received, the higher the number of citations the larger the node. If two nodes were connected, they were connected by lines, and the more nodes connected, the stronger the connection.

VOSviewer was used to analyse the clustering of co-cited literature. In WOSCC (Figure 8A), the important co-cited articles were divided into five clusters. Strong links were formed between clusters within clusters. The top 4 citations were all clustered into cluster 2, while the 5th ranked literature was clustered into cluster 1. Sakai D (2003), this node had the strongest total link strength in the network, indicating its importance. And in Scopus, it was also clustered into 6 clusters (Figure 8B). The two most co-cited articles were clustered into cluster 2, which also had the highest total link strength and played an important role in the co-cited literature network.

### Analysis of keywords

**FIGURE 9:**
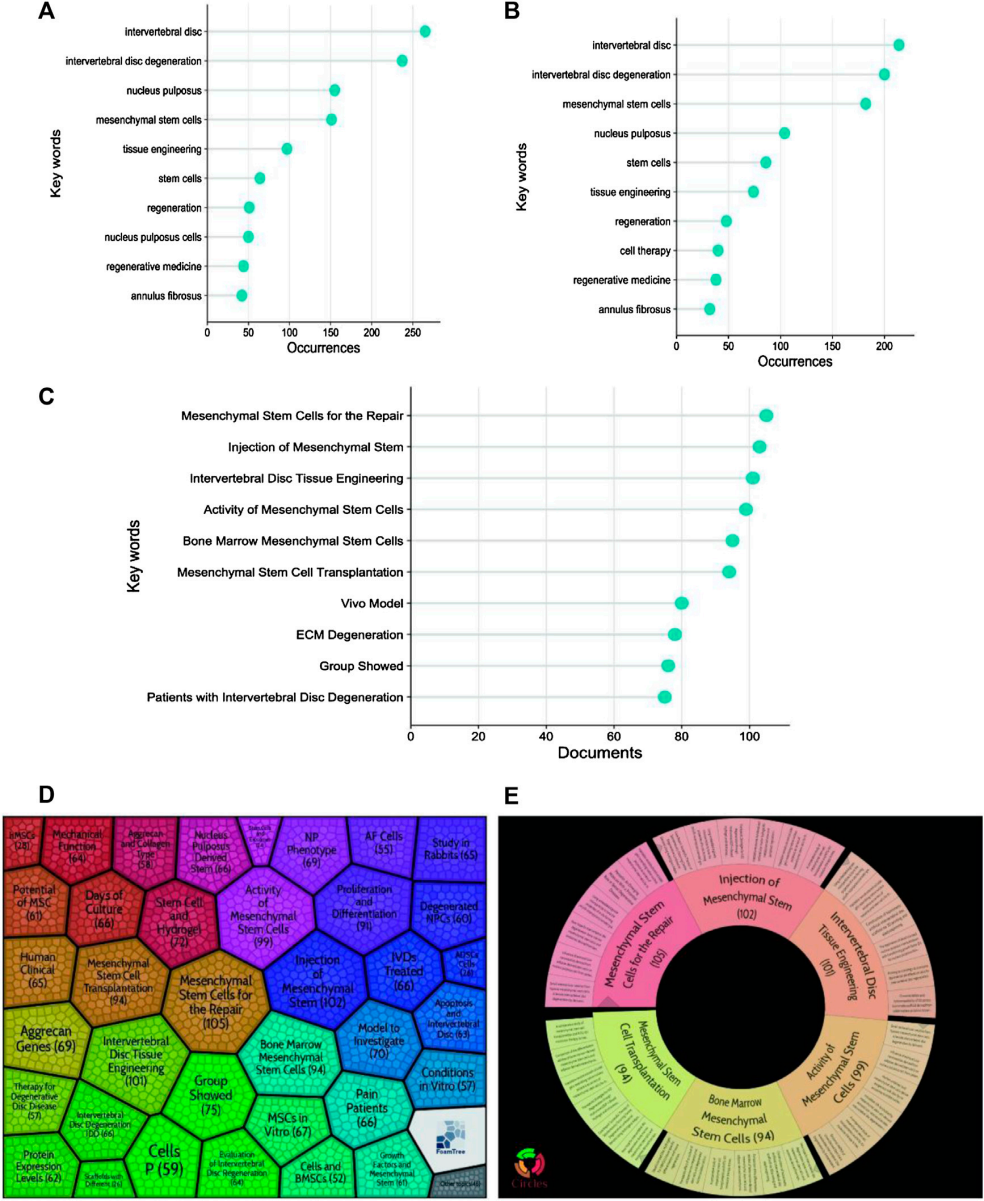
Keyword occurrence frequency statistics. **(A)**: Top 10 keywords lollipop chart in WOSCC; **(B)**: Top 10 keywords lollipop chart in Scoups; **(C)**: Top 10 topics lollipop chart in Pubmed; **(D)**: Main topics treemap using Carrot2; **(E)**: Main topics pie-chart using Carrot2.

The main keywords are listed in Table 6. As can be noticed, the top 10 keywords in terms of occurrences were mostly the same in WOSCC (Figure 9A) and Scopus (Figure 9B). They were mainly related to intervertebral disc, mesenchymal stem cells, nucleus pulposus, tissue engineering, regeneration and others. We had used Carrot2 to predict the main topics in this field, with Mesenchymal Stem Cells for the Repair appearing most frequently, followed by Injection of Mesenchymal Stem, Intervertebral Disc Tissue Engineering and Activity of Mesenchymal Stem Cells (Figures 9C–E).

**TABLE 6:** Top 10 Keywords in the WOSCC, Scoups and Pubmed database.

Rank	WOSCC		Sscopus		Pubmed	
	Occurrences	Keywords	Occurrences	Keywords	Documents	Topics
1	265	intervertebral disc	214	intervertebral disc	105	Mesenchymal Stem Cells for the Repair
2	237	intervertebral disc degeneration	200	intervertebral disc degeneration	103	Injection of Mesenchymal Stem
3	155	nucleus pulposus	182	mesenchymal stem cells	101	Intervertebral Disc Tissue Engineering
4	151	mesenchymal stem cells	104	nucleus pulposus	99	Activity of Mesenchymal Stem Cells
5	97	tissue engineering	86	stem cells	95	Bone Marrow Mesenchymal Stem Cells
6	64	stem cells	74	tissue engineering	94	Mesenchymal Stem Cell Transplantation
7	51	regeneration	48	regeneration	80	Vivo Model
8	50	nucleus pulposus cells	40	cell therapy	78	ECM Degeneration
9	44	regenerative medicine	38	regenerative medicine	76	Group Showed
10	42	annulus fibrosus	32	annulus fibrosus	75	Patients with Intervertebral Disc Degeneration

**FIGURE 10:**
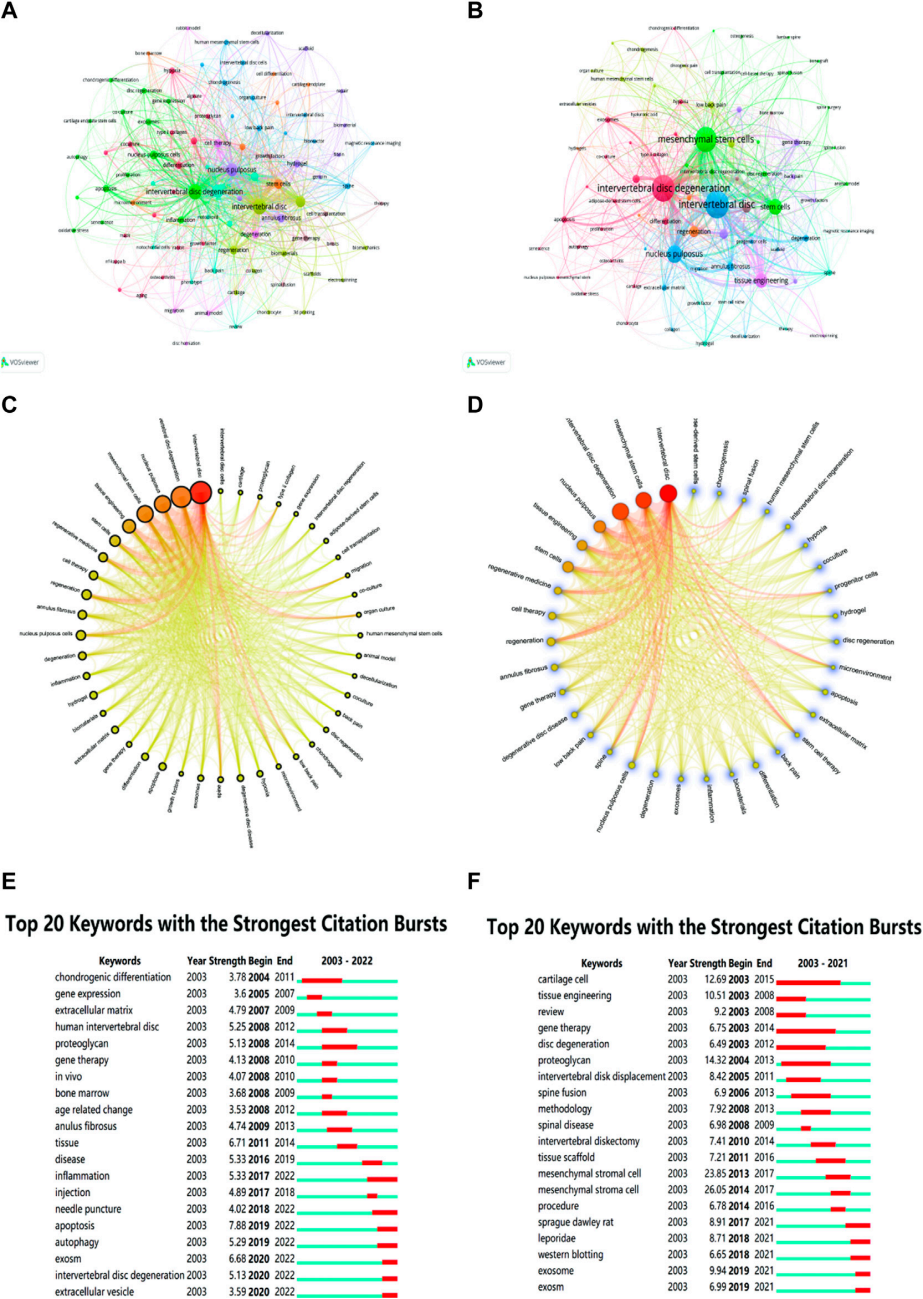
Keyword clustering, total link strength and strongest citation bursts analysis. The keywords clustering network visualization analysis in WOSCC **(A)** and Scopus **(B)**. In WOSCC and Scopus, main author keywords all formed 9 clusters. The circular diagrams **(C,D)** were used to highlight the number of major co-occurring keywords and the total link strength. The greater the occurrence frequency, the larger the node, the higher the total link strength, the redder the segment, and the larger the link between the two, the thicker and deeper the line. The top 20 keywords with the strongest citation bursts in WOSCC **(E)** and Scopus **(F)**. The color indicated the various frequent keywords (red represented frequent and green represented infrequent).

The keywords were clustered and analyzed by the VOSviewer software. In WOSCC, there were 94 keywords that co-occurred more than 5 times and were co-clustered into 9 categories (Figure 10A), while only 44 keywords that co-occurred more than 10 times. However, there were 79 keywords that co-occurred more than 5 times and were co-clustered into 9 categories, while only 35 keywords that co-occurred more than 10 times (Figure 10B) in Scopus. We applied Scimago Graphica to analyse keywords with a co-occurrence greater than 10 and presented them as a ring chart (Figures 10C,D). In the end, we used Citespace to analyse the strongest citation bursts for keywords to identify research hotspots and trends over time (Figures 10E,F).

### Clinical studies

A total of 36 eligible studies were retrieved based on topic terms. We only analysed studies that had been completed or were recruiting, filtered to obtain a total of 19 messages, and finally read the titles and content to include 6 studies (Supplementary Table S1). Three of these studies had been completed and others were recruiting patients. In terms of study design, there were four single group studies and three randomised controlled studies.

## Discussion

### Bibliometric analysis of the state of research

This article used a bibliometric approach to provide an overall assessment of the current status, hotspots and trends on the use of SCs in IDD. The results indicated that research articles had shown a year-on-year increase in recent years, suggesting that SCs were receiving increasing attention in research on the treatment of IDD. In terms of country analysis, China had the most publications, but the US had the most citations, indicating that Chinese scholars still need to enhance the quality of literature and learn from countries with higher citation rates. The analysis of the cooperative network showed that the US occupied an important position in the cooperative network and maintained close cooperation with many countries around the world, which will provide a definite boost to the development of this field. However, the total link strength of China in the cooperation network was still lacking compared with that of the US. This demonstrated that China still needs to strengthen international cooperation to promote the development of this field. The analysis of authors’ contributions showed that the international authors with high volume of publications were Sakai D, Grad S, Hoyland JA and others. It suggested that we can strengthen communication with these scholars. Although China was the first in the overall number of publications, there were fewer prominent authors, among which Zhou Y and Li FC had more publications, but the research team was still dominated by domestic collaboration and lacked international exchange. This hinted that the authors of our research still need to strengthen mutual cooperation and learn from the experience of Sakai D and other teams. The results of the journal study showed that *Spine*, *Spine Journal*, and *Biomaterials* were the journals to focus on in this field. They were recognized as key journals, not only for their large number of publications, but for their high citation rate. It was suggested that scholars can focus on the literature published in these journals when studying this area.

### Analysis of cited literature

The first literature in this field appeared in 2003 and was published in *Spine* by Gruber, HE. It was a review that focused on recent advances in intervertebral disc cell biology. In his article, he mentioned the potential of SCs in the treatment of IDD (Gruber and Hanley, 2003). The first research article was published in *Biomaterials* by Sakai, D in 2003. He transplanted mesenchymal stem cells (MSCs) embedded in Atelocollagen gel into the intervertebral disc of a model rabbit and found it to be efficient in alleviating IDD (Sakai et al., 2003). The importance of this study is evident from the fact that it is the most cited original research literature, both in WOSCC and in Scopus. In WOSCC, Chan, B. P published a review in *European Spine Journal* in 2008, which reviewed the function and methods of tissue-engineered scaffolds as an example of intervertebral discs and clarified that MSC as part of tissue engineering holds significant promise in the repair of IDD (Chan and Leong, 2008). Orozco, L published a pilot study in *Transplantation* in 2011, which was another highly cited article. He demonstrated the feasibility and effectiveness of this method by injecting autologous bone marrow mesenchymal stem cells (BM-MSCs) into the nucleus pulposus (NP) of patients with IDD (Orozco et al., 2011). This approach may become an effective alternative therapy for LDD and is widely accepted by scholars. Richardson S.M published a review in 2016 which had the highest average annual citation. The article details SCs therapy for the repair of degenerative disc, highlighting the high promise of SCs as a form of cell therapy in IDD (Richardson et al., 2016).

Co-cited references disclosed how often two articles were cited together by other articles, and this can be seen as the basis of knowledge in a specialized area (Chen, 2006). Sakai D’s articles published in *Biomaterials* in 2003 and 2006 respectively received the highest total co-citation link strength and researchers can look to these two publications for lessons (Sakai et al., 2003; Sakai et al., 2006). This article addressed for the first time that MSCs can be converted to a NP phenotype and suggests that MSCs can be used to repopulate damaged or degenerated intervertebral discs (Risbud et al., 2004). It provides a more important guide to the study of tissue engineering strategies for NP regeneration. Combining the co-citation analysis of both databases, another important paper was an original study published in 2005 in *Spine*, who demonstrated in a rabbit model that MSCs have the potential to differentiate into NP cells (Sakai et al., 2005). There were also a number of articles published before 2003 which focused on the clinical and mechanistic aspects of IDD (Buckwalter, 1995; Aguiar et al., 1999; Andersson, 1999).

### Analysis of research hotspots and research trends

Keyword co-occurrence analysis can be used to identify interests and hotspots in the field and to look for cutting-edge trends, aiding scientists to provide direction in a wide range (Zhao et al., 2021). The frequency and total link strength results in the keyword analysis indicated that MSCs were the more researched cells, with research directed mainly towards the regeneration of NP cells, which is part of tissue engineering. Using Carrot2 to analyze topic studies, the results focused on the SCs function, usage, transfer, and activity. From the above studies, it can be concluded that research was mainly divided into clinical research and mechanistic research on SCs therapy for IDD. The results of the cluster analysis of keywords also indicated that the interest of this study focused on SCs as a cell therapy for the treatment of degenerative disc disease (DDD) by regenerating NP cells. This therapy belongs to the scope of tissue engineering research. In recent years, the burst keywords have shown growing interest, which is a major indicator of the forefront trends in the sector (Wang et al., 2021).

Early research SCs therapy for IDD was associated with chondrogenic differentiation (Steck et al., 2005; Richardson et al., 2006), affecting gene expression (Vadala et al., 2008), and others. This was followed by a series of mechanistic studies to demonstrate how SCs differentiate into myeloid cells (Lu et al., 2008; Stoyanov et al., 2011) and tissue engineering scaffolding studies (Calderon et al., 2010). In recent years, there have been a number of hot topics and trends focusing on inflammation (Zhao et al., 2020; Ekram et al., 2021), apoptosis (Nan et al., 2020b; Hu et al., 2021), autophagy (Nan et al., 2020a; He et al., 2021), and exosome (Hu et al., 2021; Krut et al., 2021). However, there is no single mechanism for SCs treatment of IDD, and multiple mechanisms are often intertwined, which requires an overall grasp and further exploration.

### Clinical study analysis

ClinicalTrials.gov, as a database offered by the US National Library of Medicine, contains a collection of reference clinical trials conducted around the world on a variety of conditions and diseases (Rechberger et al., 2021). We have used the clinicalTrials website as a basis for some analysis of clinical studies. Only 6 of the registered clinical observational studies were eligible, showing that although a large amount of basic research has been conducted on SCs therapy for IDD, clinical studies are still relatively small at present and further randomised controlled studies (RCT) are needed at a later stage to improve the level of evidence. The current clinical use of SCs in the treatment of IDD is mainly by direct injection into the degenerated disc of the patient. From the clinical trials that have been screened and registered, the main types of SCs used are Human Umbilical Cord Mesenchymal Stem Cells (HUC-MSCs), BM-MSCs and Adipose Stem Cells. BM-MSCs are the most widely used. This suggests that our later clinical studies may also focus on assessing the efficacy and safety of BM-MSCs injected into degenerated discs in patients with DDD.

## Strengths and limitations

This study is the first relatively systematic bibliometric and visual analysis for SCs in IDD and provides some insight for scholars studying this field. But it still has some limitations. Although this article searched WOSCC, Scopus and Pubmed and conducted some analysis, it was independent and did not sufficiently incorporate the data. The study only included articles whose language was English, ignoring non-English articles, and only original studies and reviews were included, all of which resulted in the analysis not being fully comprehensive. The database is updated in real time, which leads to the possibility that our results may differ from those available at the time of publication. Fewer studies were included for clinical studies and there were no complete conclusions. Therefore, it remains important to keep abreast of the latest and neglected literature to gain a comprehensive understanding of current hotspots and future trends, while further research is needed in clinical research methods to clarify efficacy.

## Conclusion

This study demonstrates the global research hotspots, trends and clinical use of SCs in the treatment of IDD. Annual publication trends in this study are increasing, with China being the most published country and the US being the largest contributor, both of whom are ahead of the curve in this field. Sakai D, Grad S and Hoyland JA have made outstanding contributions and are recognized by other scholars for their high productivity and the quality of their research. *Spine* is the most published and most cited journal, in addition to *Spine Journal* and *Biomaterials*, which are also more authoritative journals and have received high citations. All of them have received high citations. Keyword co-occurrence studies suggest that the current hotspots are in mechanistic studies, including inflammation, apoptosis, exosome, and autophagy. Some studies have also investigated tissue-engineered scaffolds of SCs to better repair degenerated discs. Clinical studies are relatively scarce. Direct injection of MSCs into degenerated discs for the treatment of DDD is the current direction of research, but large, multicentre clinical studies are still needed to demonstrate the efficacy, and safety of this method.

## Data Availability

The raw data supporting the conclusions of this article will be made available by the authors, without undue reservation.
