# Supplementary material for: Global research status and hot trends in stem cells therapy for Intervertebral disc degeneration: A bibliometric and clinical study analysis
Source: Front Pharmacol. 2022 Aug 8;13:873177. doi: 10.3389/fphar.2022.873177 (PMC9393636; doi:10.3389/fphar.2022.873177)
Supplement: Supplementary file 1 [file Table1.doc]

**TABLE S1 |** Clinical studies in “ClinicalTrials.gov” about this field

| **Rank** | **Status** | **Study Title** | **Disease** | **Intervention** | **Period** | **Patient numbers** | **Country** |
| --- | --- | --- | --- | --- | --- | --- | --- |
| 1 | Recruiting | Human Umbilical Cord Mesenchymal Stem Cells For the Treatment of Lumbar Disc Degeneration Disease | Lumbar Disc Herniation | Human Umbilical Cord Mesenchymal Stem Cells | 2020.08-2022.03 | 20 | China |
| 2 | Completed | Treatment of Degenerative Disc Disease With Allogenic Mesenchymal Stem Cells (MSV) | Degenerative Disc Disease | Biological: Allogenic Mesenchymal Stromal Cells|Drug: Mepivacaine | 2013.04-2017.04 | 25 | Spain |
| 3 | Completed | Adipose Cells for Degenerative Disc Disease | Degenerative Disc Disease | Procedure: Adipose Stem Cells | 2014.03-2017.01 | 15 | USA |
| 4 | Completed | Clinical Trial Based on the Use of Mesenchymal Stem Cells From Autologous Bone Marrow in Patients With Lumbar Intervertebral Degenerative Disc Disease | Intervertebral Disc Disease | Procedure: Instrumented posterolateral arthrodesis | 2010.01-2017.05 | 15 | Spain |
| 5 | Recruiting | Effectiveness and Safety of Mesenchymal Stem Cell (MSC) Implantation on Degenerative Discus Disease Patients | Degenerative Disc Disease | Drug: Mesenchymal Stem Cell + NaCl 0,9% 2ml | 2017.07-2020.12 | 10 | Indonesia |
| 6 | Recruiting | Efficacy of Intradiscal Injection of Autologous BM-MSC in Worker Patients Affected by Chronic LBP Due to Multilevel IDD | Intervertebral Disc Degeneration | Drug: Autologous BM-MSC|Other: Sham Procedure | 2020.11-2021.07 | 52 | Italy |
